# Supplementary material for: Agreement threshold on Axelrod’s model of cultural dissemination
Source: PLoS One. 2020 Jun 2;15(6):e0233995. doi: 10.1371/journal.pone.0233995 (PMC7266322; doi:10.1371/journal.pone.0233995)
Supplement: Attachment — Submitted filename: PlosOne_review_2019.pdf [file pone.0233995.s001.pdf]

PLOS ONE Review

Agreement Threshold on Axelrod's model of Cultural Dissemination

This manuscript examines the horizontal transmission of traits through a population by modifying a classic agent-based model of social transmission (Axelrod 1997) in such a way that it more accurately reflects the transmission of ordinal or continuous traits typically measured empirically through social surveys. In addition, the authors present some novel visualization techniques for both the output of their model and for empirical survey data.

I have several concerns about this manuscript that I would like to see addressed before I can recommend it for publication.

1) The code to run the simulations and to produce the novel plots presented in the manuscript is not made available. No one can evaluate the results of the model without the code. Importantly, the new plotting techniques are a potentially valuable contribution of the manuscript. However readers interested in using these methods are forced to figure out how to implement them on their own. I would recommend writing the ABM and the plotting scripts in R, as this would make these innovations accessible to a broad range of researchers from different disciplines.

2) I found Figure 1 to be confusing. Because nodes are connected with lines representing their similarity, it was difficult for me to discern the boundaries between clusters. Instead, I would recommend using lines in the spaces around nodes (rather than lines connecting nodes) to represent similarity/difference, and presenting a time series of plots so that readers can see the clusters develop over time. In other words, model your figure after the original Axelrod (1997) Figure 1.

3) I don't understand the interpretation given for the dispersion parameter d in equation 1. It seems that d is equivalent to n in equation 9 of the cited reference (Duffuant 2006), which is the "generalized number of clusters" according to this reference. However, in the manuscript it is stated that the inverse of d represents the generalized number of clusters (lines 154-155). Similarly, it is stated that Figure 6 plots d, which does seem to be the inverse of the number of clusters. Am I correct that what you have actually plotted in Figure 6 is $1/d$?

4) I don't understand the explanation for why adding an agreement threshold a results in an increasing number of clusters at equilibrium as the number of features (F) increases. The explanation given on lines 160-162 is: "As the number of features increases, there are a larger number of feature-trait combinations an agent can have. For a low agreement threshold an agent would not be able to interact with any of its neighbours." If I understand correctly

what is written, this process serves to increase the number of isolated nodes at equilibrium, which in turn contributes to a larger number of clusters. However, this runs counter to my intuition. It's quite possible that I have misunderstood something about the model, so I will outline my thinking below. If there is some error in my logic, perhaps the authors would consider including some additional explanation in the paper so that readers don't make similar mistakes.

One of the important insights from the model of Axelrod (1997) is that, for a given number of traits per feature, increasing the number of features (F) will increase the chance that two neighboring nodes have the same trait on at least one feature, thereby increasing the chance that they interact, and resulting in an overall decrease in the number of stable clusters at equilibrium. In contrast, for a given number of features, increasing the number of traits per feature (q) will decrease the probability that two neighboring nodes share the same trait on any one feature, thereby decreasing the chance that they interact, and resulting in an overall increase in the number of stable clusters at equilibrium (see Axelrod 1997, pg 212-213).

This can be shown mathematically. For instance, consider a system with two features ($F = 2$) and three traits per feature ($q = 3$). Any given node can take on one of nine possible trait combinations across its two features (e.g., $(F_1 = 1, F_2 = 1), (F_1 = 1, F_2 = 2), ..., (F_1 = 3, F_2 = 3)$). For any given trait combination, there are four of these nine trait combinations that a neighboring node may take on which would render interaction between these nodes impossible. For instance, if the focal node is $(1, 1)$ and the neighboring node is $(2, 2)$, interaction is impossible because the nodes do not share traits on either feature. From this, we can calculate the probability that two nodes cannot interact as: $Pr(\text{no interaction}) = (\frac{1}{9} \cdot \frac{4}{9}) \cdot 9 = \frac{4}{9} = 0.44$. If we increase the number of features to three ($F = 3$, $q = 3$), these calculations become: $Pr(\text{no interaction}) = (\frac{1}{27} \cdot \frac{8}{27}) \cdot 27 = \frac{8}{27} = 0.30$. Therefore, adding a feature has increased the probability that two nodes will interact from $1 - 0.44 = 0.56$ to $1 - 0.3 = 0.7$. In general, the probability that two nodes can interact is $Pr(\text{interaction}) = 1 - \left(1 - \frac{1}{q}\right)^F$. If my generalization is correct, then one can see that increasing q will decrease this probability, while increasing F will increase it, as Axelrod (1997) concluded.

The innovation introduced in this manuscript is to add an agreement threshold, a (an integer), to the model. As I understand manuscript lines 118-122, this means that two nodes can interact if the trait for at least one feature of the focal node is within a units of the neighboring node's trait for that feature. Thus, for a system with one feature, three traits, and an agreement threshold of one ($F = 1$, $q = 3$, $a = 1$), given that the focal node has trait 1, interaction can occur if the neighboring node has trait 1 or 2, but not if it has trait 3. If the focal node has trait 2, interaction will occur if the the neighboring node has trait 1, 2, or 3. For an analogous system with two features ($F = 2$, $q = 3$, $a = 1$), if the focal node has $(1, 1)$, then interaction is prevented only if the neighboring node has $(3, 3)$. If the focal node has $(1, 2)$, then interaction will always occur regardless of the traits of the neighbor,

because any trait that the neighbor has for feature two is within the agreement threshold for the focal nodes's trait for feature two. There are four trait combinations (out of nine possible) that the focal node can have ($(1,1)$, $(1,3)$, $(3,1)$, or $(3,3)$) which would result in non-interaction if the neighboring node has a certain trait combination ($(3,3)$, $(3,1)$, $(1,3)$, or $(1,1)$, respectively). For all other trait combinations of the focal node, interaction will always occur. Thus, in this system, the probability of no interaction is $4 \cdot \left(\frac{1}{9} \cdot \frac{1}{9}\right) = \frac{4}{81} = 0.049$. Adding an additional feature ($F = 3$, $q = 3$, $a = 1$) results in the following calculations: $6 \cdot \left(\frac{1}{27} \cdot \frac{1}{27}\right) = \frac{6}{729} = 0.008$. Therefore, adding a feature has increased the probability that two nodes will interact from $1 - 0.049 = 0.95$ to $1 - 0.008 = 0.99$. One can see that adding an agreement threshold to the model, even the lowest possible threshold ($a = 1$), substantially increases the probability that two nodes will interact, relative to the base model with no agreement threshold. Furthermore, it appears that the insight from the original Axelrod model is preserved: increasing the number of features, increases the probability that two nodes will interact. In the original model, this served to decrease the number of clusters at equilibrium. However, in the manuscript, increasing the number of features seems to increase the number of clusters. I don't understand why. As I believe I've shown, the fact that increasing the number of features increases the number of feature-trait combinations that a node can have, does not, in fact, decrease the probability that a node will interact with its neighbors (contra lines 160-162).

In summary, I believe the authors should more fully explain the reason why increasing the number of features increases the number of clusters in the model, given a low agreement threshold.

Axelrod, R (1997) The dissemination of culture: a model with local convergence and global polarization. Journal of Conflict Resolution 41(2):203-226.

Deffuant G (2006) Comparing extremism propagation patterns in continuous opinion models. Journal of Artificial Societies and Social Simulation 9(3):8 `http://jasss.soc.surrey.ac.uk/9/3/8.html`
