# Supplementary material for: Agreement threshold on Axelrod’s model of cultural dissemination
Source: PLoS One. 2020 Jun 2;15(6):e0233995. doi: 10.1371/journal.pone.0233995 (PMC7266322; doi:10.1371/journal.pone.0233995)
Supplement: Attachment — Submitted filename: response.pdf [file pone.0233995.s002.pdf]

1. I think there is insufficient consideration of the existing literature, which is rather extensive for the Axelrod model and various enhancements or modifications of it. There are some reviews of this literature, and two are cited (citations [28] and [29], Flache et al. (2017) and Castallano et al. (2009)), but these reviews are not necessarily exhaustive of the (very extensive) literature on the Axelrod model (and the longer review article is 10 years old).

Reply: Thanks for this, we have added more references and expanded the introduction (in red) and are happy to add more or expand more on this if necessary.

In particular, although some of the Flache & Macy arXiv preprints introducing it are cited, I would have expected more discussion of "bounded confidence" as it is so closely related to the "agreement threshold" idea, and in particular, De Sanctis & Galla (2009) and Hegselmann & Krause (2002) are good sources for this (the latter is not about the Axelrod model, but gives some citations for the history of the "bounded confidence" idea in other models). Another very useful paper on the Axelrod model is Flache & Macy (2011), who state that:

"The bounded confidence models showed that global diversity does not depend on the assumption that opinions are discrete, as long as influence can only occur between individuals who are sufficiently similar. Thus, while Axelrod introduced two innovations—discrete opinions and homophily — these subsequent studies showed that the latter is sufficient for local convergence to preserve global diversity."
(Flache & Macy (2011), p. 972).

These papers are all cited in the Flache et al. (2017) review, which discusses bounded confidence, but I think it is necessary to do more than just cite the review in this manuscript, and actually also directly cite the individual papers and discuss bounded confidence and its relationship to the "agreement threshold".

Reply: Bounded confidence was the main reason we came up with this model so was a big influence, however, we removed this after multiple discussions from those mentioned in the acknowledgements. Their argument was that with the traits and bound not being continuous it was too confusing to call it bounded confidence when we were dealing with discrete values – even though there are models out there that take discrete values. Also you'll see at one point we discuss something about not using continuous values, the survey discussion was added in after to convince them that taking discrete values had merit. Initially this paper was framed more theoretically in terms of adding this bounded confidence extension (which we later renamed an agreement threshold), we added the survey framing after their comments.

2. The main motivation is to use survey data with ordinal scales (e.g. Likert scales) in the Axelrod model. However (related to point 1 above) this has already been done in published literature, which is not cited in the manuscript. Specifically, Valori et al. (2012) use an Axelrod model with empirical data from surveys as the initial culture vectors (rather than the usual random initialization), as do some subsequent works, for example Babeanu et al. (2017) [see Appendix A for details of treatment of survey data with nominal and ordinal cultural features]; Babeanu et al. (2018); Stivala et al. (2014).

The details of this in the Valori et al. (2012) paper are largely in the Supplementary Information, but they are there (see S.I. p. 3), showing how both nominal and ordinal features are treated in their version of the Axelrod model. Even some of the "further work" in the manuscript such as "vary q per feature" is already implicitly described there.

In addition, the Valori et al. (2012) S.I. discusses the arbitrariness of the number of features F and how bounded confidence can compensate for this (see SI. pp. 6-7 and footnote on p. 7), which was a motivation in the manuscript for introducing the agreement threshold.

Reply: Many thanks for this, though familiar with the paper I confess I had not looked at the SI. We have added this to the introduction now and again are happy to elaborate further if necessary.

3. Similarly, although I am not familiar with any previous publications using the bipartite graph idea, the one-mode projection where nodes are agents would appear to be a weighted version of the "cultural graph" described by Valori et al. (2012), in which an edge exists between agents with sufficiently similar (according to the value of the confidence threshold) culture vectors. Although in Valori et al. (2012) and subsequent work building on it the "cultural graph" was not used for visualization, it nevertheless was defined and used for analysis of the model.

Reply: We have added a sentence on this.

4. I am not convinced of the utility of the bipartite graph visualization (e.g. Fig. 2). It seems to me that the presence of the agents as nodes and the density of lines showing the feature-trait combinations really just clutters the visualization (and would quickly become unreadable for larger values of N, q, and F). Would it not be simpler just to represent the relative proportions of the feature-trait combination as a heat map, for example? Valori et al. (2012) visualize correlations between opinions as heat maps and dendrograms (as they are interested in the structure of such correlations), but the simple proportions of each trait could also be visualized directly this way.

Reply: For pure Axelrod as the feature-trait combination are arbitrary it can look strange, however increasing in size won't be a problem as the brightness increases. However, out main interest in this type of visualisation will come when we actually apply it to survey data, see this image for example:
https://www.dropbox.com/s/d8zv783gf4ijy19/attitudes_net_2113_kk.png?dl=0
The two types of nodes are people and their responses to eight questions, as the features (questions) and traits (responses) are no longer arbitrary we think it's a nice way to visualise this type of data which is why we included it here. We decided we wanted to keep this paper theoretical though so did not add the empirical data.

5. Although I believe the "agreement threshold" is a new and useful contribution, I found its explanation a little confusing at first. I think it might be clearer to specify it not just in English but also precisely in mathematical notation, as is done in the physics literature on the Axelrod model for describing the measure of cultural similarity etc. In the abstract (where mathematical notation is inappropriate) on a first reading it seemed to me that the "agreement threshold" was just the same as "bounded confidence", and it required reading the manuscript carefully to see that it is not.

Reply: We have added an equation to the section now.

6. On p. 2 "Specifically, when the number of features F is greater than the number of traits q , usually there will be consensus with only one cluster emerging [8]". I'm not sure that this statement is supported by citation [8] (Castellano et al. (2000)). [See point (2) above for more on how the arbitrariness of the number of features F has previously been handled for survey data using bounded confidence.]

Reply: Perhaps this is better phrased as multicultularity is found for very high numbers of q are required but in out work we have a small value (5 or 7 typically). We have edited this and added that bounded confidence was suggested as a fix for this.

7. Why is the number of clusters counted with the "dispersion indicator" rather than a more conventional order parameter for the Axelrod model such as the mean size of the largest cluster, or simply the number of cultures?

Reply: We initially used cluster size but were recommended the dispersion indicator, however we are happy to go back to number of clusters. We went with the mean (of 1000 simulations) and rather than use standard deviation or the 5 and 95 percentile, we instead show another figure with the density plot of a specific q and F varying the agreement threshold to get an idea of effect of the changing parameters and the distributions are clearer to see than with error bars in the plot for the means. (Note we have done it with the error bars but feel there is a bit too much going on, see:
https://www.dropbox.com/s/c2x1gb5x2nj3phs/n_clusters_95_percentile.png?dl=0
however, we are happy to put this in also if necessary).

There is a mention of phase transitions as further work in the Conclusions section, and indeed no phase transition is apparent in Fig. 6. Presumably this is because the quantity on the x axis does not control a phase transition (while q does, for example). Or perhaps is the "dispersion indicator" actually not an order parameter?

Reply: We removed the dispersion index as, although we were recommended to use it, we didn't really understand it clearly. We also removed mention of a phase transition.

8. I found Figure 6 (or more precisely its axis labeling) confusing. What is the x axis exactly? It is labeled "No. features" so it seems to be the dimension of the culture vector i.e. F. If so I think it would be better to clearly state this. [There is also a typo in the caption, should be q = 7 not q - 7].

Reply: Thanks, we have edited this now.

9. I don't think the computational experiments are thorough enough. In the absence of any analytical treatment (mean-field approximation for example) simulation experiments are all that can be done, which is reasonable. However the only computational experiments seem to be those represented in Figure 6 showing the dispersion indicator in the absorbing state (final state) for two values of q (and the corresponding possible values of a) and one value of N. There are no error bars so it would seem these represent just a single run of the model for each parameter combination. At a minimum I would expect the results of multiple runs (from random initial conditions) and the figure then showing the mean and standard deviation (or 95% confidence interval) over many runs, as is done in the vast majority of publications on Axelrod model variants.

Reply: We have changed to the mean number of clusters and provide the density plots as well as do this for both N = 100 and N = 1024 now. Also we have made it more clear that each point represents 1,000 simulations and expanded on the discussion in that section.

10. The manuscript defines a bipartite network as "a network with two types of nodes" (p. 2) but omits the essential point that any edge must be between nodes of two different types (never two nodes of the same type). Indeed a bipartite graph is conventionally defined as a graph in which the nodes can be partitioned into two disjoint sets such that every edge connects a node in one set to a node in the other.

Reply: We have put in that edges have to connect different types of nodes now to clarify this.

11. Although PLOS ONE requires data be made freely available, it appears to have no such requirement for code. Hence I cannot insist that the code for the modified Axelrod model with agreement threshold be made available, nevertheless I think it would be preferable to do so to facilitate reproducbility. Even a "toy" version in e.g. NetLogo would allow people to verify their undertanding of the model description in the manuscript (see also point (5) above) or compare their own implementation to check they match.

Reply: The code was written in Python so I have uploaded a script to my github and linked at the end.

Reviewer #2

1) The code to run the simulations and to produce the novel plots presented in the manuscript is not made available. No one can evaluate the results of the model without the code. Importantly, the new plotting techniques are a potentially valuable contribution of the manuscript. However readers interested in using these methods are forced to figure out how to implement them on their own. I would recommend writing the ABM and the plotting scripts in R, as this would make these innovations accessible to a broad range of researchers from different disciplines.

Reply: The code was written in Python so I have uploaded a script to my github and put the link at the end.

I found Figure 1 to be confusing. Because nodes are connected with lines representing their similarity, it was difficult for me to discern the boundaries between clusters. Instead, I would recommend using lines in the spaces around nodes (rather than lines connecting nodes) to represent similarity/difference, and presenting a time series of plots so that readers can see the clusters develop over time. In other words, model your figure after the original Axelrod (1997) Figure 1.

Reply: The reason we chose to do it this way is on the next figure we want to represent a node as an agent in the bipartite graph. Then there is a connection between that node and its attitude, if we use the “site” which is what is traditionally done then it is hard to translate that to a node when connecting it to the attitude. We have put in a statement on this hopefully making it less confusing.

3) I don't understand the interpretation given for the dispersion parameter d in equation 1. It seems that d is equivalent to n in equation 9 of the cited reference (Duffuant 2006), which is the "generalized number of clusters" according to this reference. However, in the manuscript it is stated that the inverse of d represents the generalized number of clusters (lines 154-155). Similarly, it is stated that Figure 6 plots d, which does seem to be the inverse of the number of clusters. Am I correct that what you have actually plotted in Figure 6 is 1/d?

Reply: We have changed to the mean number of clusters and provide the density plots as well as do this for both N = 100 and N = 1024 now. Also we have made it more clear that each point represents 1,000 simulations and expanded on the discussion in that section. We hope that this has made that section clearer.

4) I don't understand the explanation for why adding an agreement threshold a results in an increasing number of clusters at equilibrium as the number of features (F) increases. The explanation given on lines 160-162 is: "As the number of features increases, there are a larger number of feature-trait combinations an agent can have. For a low agreement threshold an agent would not be able to interact with any of its neighbours." If I understand correctly what is written, this process serves to increase the number of isolated nodes at equilibrium, which in turn contributes to a larger number of clusters. However, this runs counter to my intuition. It's quite possible that I have misunderstood something about the model, so I will outline my thinking below. If there is some error in my logic, perhaps the authors would consider including some additional explanation in the paper so that readers don't make similar mistakes.

Reply: The agreement threshold is used in the copying of traits as well as in the calculation of a probability for interaction.

The probability for interaction between two nodes is increased when the agreement threshold is included (vs standard Axelrod) as features in which you are (for example) 1 trait away from each other now add to the probability for interaction. However, once you decide interaction can occur, the only features you can change are the ones in which the traits are sufficiently similar (within the agreement threshold). In standard Axelrod once interaction happens one feature will be copied, thereby increasing the probability of interaction in the future. In our version you only change the features which are already quite similar, meaning the probability for interaction in the future is the same.

We have added something on this hopefully clarifying it further but are happy to add more if necessary.

Reviewer #3

It is unclear if the method for displaying the system state as a bipartite graph provides any benefit. For instance, the conclusions they reach through Figure 2 would be easier to observe through a usual frequency plot. Furthermore, the figures provide a poor visualization of the bipartite graph and it is difficult to see where are the traits nodes. If there was some benefit in the bipartite representation they

should make use of this representation to achieve something that would not be possible without a network, e.g., finding communities or computing distances. This is not the case in this paper.

Lastly, their modification to the Axelrod model is not explored seriously. Results are analysed in terms of individual executions without any clear demonstration of how representative they are. Fig. 6 seems to be the only one corresponding to ensemble averages, however, it lacks statistical information.

Reply: We have changed to the mean number of clusters and provide the density plots as well as do this for both N = 100 and N = 1024 now. Also we have made it more clear that each point represents 1,000 simulations and expanded on the discussion in that section.

Specific remarks

- Authors refer to the paper for attitudes and response-options, but it is not clear how they are related to features or traits. Do attitudes correspond to the number of traits or features?

Reply: We mean the combination, if on a survey your response to question 1 is: 3, then your attitude is question1-response3.

- To verify the number of clusters in the simulation, the authors use the dispersion indicator. However, if the goal is to count the number of clusters, wouldn't be more natural to check directly the number of clusters?

Reply: We have changed to the number of clusters and provide the density plot to visualise the distribution.

- Some phrases of the manuscript are really hard to understand and the authors should work on the writing, e.g., line 180.

Reply: Line 180 for us is in the acknowledgements, however we have cleared up many sentences as highlighted in the text.
