# Supplementary material for: Agreement threshold on Axelrod’s model of cultural dissemination
Source: PLoS One. 2020 Jun 2;15(6):e0233995. doi: 10.1371/journal.pone.0233995 (PMC7266322; doi:10.1371/journal.pone.0233995)
Supplement: Attachment — Submitted filename: response.pdf [file pone.0233995.s003.pdf]

Reviewer #3: 1. I believe the authors have not provided an appropriate response to the comments made by myself and by reviewer 1 about the usefulness of the graph bipartite representation. According to their comment on line 93: "For example, we observe that features 1 and 2 both have a majority but feature 3 is split between q = 1 and q = 4. This is particularly useful if using this network to represent survey data. If there are three questions with a five-scale response, it is clear which response is the most favoured per question." The authors are reaching this conclusion through the degree of the nodes in the graph plot, which would be a lot clearer in a simple plot showing the frequency of traits per feature. Besides this, I don't see any benefit provided by the bipartite representation and don't believe it is a contribution of the paper. For it to be a contribution, the authors should have used it for some analysis that needed the network representation. Now, it seems to be a rather unnecessary artefact.

Reply: We have added in a paragraph about taking projections from the bipartite representation which is what the figures with clusters represent to show that this is relevant later, for example figures 4 and 5 are projections from the bipartite graph in figure 3. This is subtly different to cultures in standard Axelrod (see response to point 2 for more details) as here a cluster represents groups of people who fully agree, they do not need to be separated. In figure 1 if the lower left and upper right regions have the same traits for each feature in standard Axelrod then they would be two cultures, using the bipartite projection, they would be one opinion-based group. We have added text to clarify this.

We appreciate from a model point of view this can look strange, but from an empirical point of view this is a different way of viewing survey data. For example, the image we mentioned to Reviewer #1 in the first review:
https://www.dropbox.com/s/d8zv783gf4ijy19/attitudes_net_2113_kk.png?dl=0
shows a survey with two types of nodes being the participants and their responses to eight questions, as the features (questions) and traits (responses) are no longer arbitrary we think it's a nice way to visualise this type of data which is why we included it here. We decided we wanted to keep this paper theoretical though so did not add the empirical data.
This is something from our upcoming empirical paper which we do not really want to include here, we would also like to use this agreement threshold model on empirical data after which is why we would rather leave this bipartite visualisation in.

2. Still regarding the bipartite representation, the authors mention in the abstract: "This visualisation is particularly useful when representing survey data as it illustrates the coevolution of cultures and opinion-based groups in Axelrod's model of cultural diffusion." In the introduction, however, authors mention: "Opinion-based groups (or "cultures") are formed by people holding a particular selection of attitudes." It seems, thus, that cultures and opinion-based groups correspond to the same concept. What would they refer to when discussing about coevolution? Furthermore, it is unclear how this visualization illustrates the supposed coevolution as it is just a representation of the stationary state.

Reply: This is an important point that we didn't properly distinguish between. In Axelrod (1997), a culture in the model is a group of agents sharing the same traits for each feature that are physically next to one another. Here, however, as we take a projection from the bipartite graph, the cluster of those who agree entirely are an opinion-based group even if they are physically separated. We have made this more clear in the text by including the line:

We remove this spatial constraint when identifying clusters and instead get groups of agents sharing the same traits for each feature, we refer to these as *opinion-based groups*.

3. Intuitively, it makes sense the use of the agreement thresholds for computing the probability of interaction between agents. Nonetheless, its use in the process of social influence, i.e., copying traits of features that are in the agreement threshold, seems rather unnatural. The authors should add some reference justifying why social influence should only occur in features that are a traits away. Furthermore, this characteristic of the model should be the motive it does not reach consensus for small q and a, as consensus can be unreachable from initialization.

Reply: Thank you for pointing this out. We had previously included references justifying this feature of social influence, that have now been re-added in the introduction. Further justification is that people tend to display bias in evaluating evidence by favouring views that correspond to their existing attitudes (Lord, ross & Lepper, 1979; Reedy, Wells & Gastil, 2014). For example, empirical research shows that people rate arguments as more compelling when they correspond to previously held attitudes (Taber & Lodge, 2006). The mechanism at play may be biased information processing (i.e. confirmation bias), rather than social influence per se. Either way, we believe it should be accounted for in models of opinion sharing, as indeed it is in many others (Deffuant et al., 2000; Hegselmann et al., 2002). A section similar to this has been added to the introduction.

Taber, C. S., & Lodge, M. (2006). Motivated skepticism in the evaluation of political beliefs. American Journal of Political Science, 50 (3), 755–769.

Reedy, J., Wells, C. and Gastil, J. (2014), How Voters Become Misinformed: An Investigation of the Emergence and Consequences of False Factual Beliefs*. Social Science Quarterly, 95: 1399-1418. doi:10.1111/ssqu.12102

Lord, C. G., Ross, L., & Lepper, M. R. (1979) Biased assimilation and attitude polarization: The effects of prior theories on subsequently considered evidence. *Journal of Personality and Social Psychology, 37*(11), 2098–2109. https://doi.org/10.1037/0022-3514.37.11.2098

Deffuant G, Neau D, Amblard F, Weisbuch G (2000), Mixing beliefs among interacting agents. Advances in Complex Systems. 01n04), 7–98.

Hegselmann R, Krause U, et al. (2002), Opinion dynamics and bounded confidence models, analysis, and simulation. Journal of artificial societies and social simulation. 5,3.

4. In the conclusions, the authors mention: " The clustering that emerges is useful for considering opinion-based groups in opinion dynamics models and empirical data such as surveys. " It is unclear how the clusters found in this model can be useful for the applications mentioned. The authors should discuss this more thoroughly.

Thank you for identifying this weakness in our explanation. We have strengthened the introduction in two ways. 1) We have made it clearer that the Axelrod model is a non-parametric opinion model easily adapted to ordinal data such as Likert-type scale responses and that this approach has important advantages over alternatives (e.g. averaging models). 2) We clarify that monocultural states are almost never seen in survey data (and such lack of variability is actively avoided when designing survey questions). For this reason the vanilla Axelrod model is not at all useful for modelling survey data, since as soon as the number of features (ie. survey items) exceeds the number of traits (ie. response options) homogeneity is guaranteed in a fully connected topology.

Specifically:

We have edited this sentence in the introduction, adding the highlighted text: “In this paper we demonstrate that an adapted version of Axelrod’s model of cultural dissemination [7] can be used to model opinion-based groups with data structures similar to survey data.”

We have edited the following sentence and added two following sentences, adding the highlighted text: “In principle, conceptualising Axelrod’s nominal cultural features and traits as ordinal attitudes will allow us to model the emergence of opinion-based groups with a data structure that maps cleanly on to raw survey data.” Note that opinion surveys typically use ordinal Likert-type response items (e.g. an item with several response options from Strongly Disagree to Strongly Agree). While these are frequently treated as interval data in analyses (ie. assuming consistent intervals between scale points and allowing arithmetic operations such as addition and subtraction), there is a strong argument that individual Likert-type items should properly be treated as ordinal, allowing only non-arithmetic operations. The original Axelrod model of cultural dissemination relies only on swapping, and our adaptions adds ranking, making it an excellent fit with Likert-type data.

We have changed the following sentence: “The clustering that emerges is useful for considering opinion-based groups in opinion dynamics models and empirical data such as surveys.”

Instead we argue:

"The resulting model is suited to natively modelling survey data, which frequently consists of Likert-type responses, since only ranking and swapping operations are required thus respecting the ordinal nature of the data. The model also avoids the homogoneous end-state which would always result when the original Axelrod model is used on survey-type data, as there are almost always features than traits (in other words, more items than response options).”

5. On line 192: "One outcome of this model is that extremists are less likely to change their position than moderates." This is not an outcome of the model, this is given by the specification of the model. If agents cannot copy the features which are not in the agreement threshold, features with extreme value traits cannot be modified unless the agent's neighbours features are also in the same extreme.

Reply: Indeed this is a specification rather than an outcome, and we have rephrased this sentence accordingly.

Specific remarks:

- The authors update the definition of the bipartite network according to the comment of reviewer 1, however, the definition still is not precisely correct.

Reply: Changed to "a graph with two types of nodes where edges must connect nodes of different types"

- What is the total number of agents, F, q in the simulations of figure 5?

Reply: F=6 and q=3, this is one of the two projections of the of figure 3 (this has been added to the caption).

- "A further extension could specify that if the agreement threshold is larger than one, the traits move towards each other rather than one agent copying the other." This actually could be used without the restraint posed by the initial condition of the model, i.e., of only copying features which are inside the agreement threshold. In my opinion, this makes more sense than the current approach.

Reply: Our model preserves those at the extremes whereas a model that allows both people move towards each other finds everyone ending up in the middle or the spectrum (See ref 36. Flache et al 2017 table 1). This prevalence of those on extremes is something that is observed in real systems (Brandt at al., 2015), we have added this reference now.

Brandt at al (2015). The Unthinking or Confident Extremist? Political Extremists Are More Likely Than Moderates to Reject Experimenter-Generated Anchors. Psychological Science, 26(2), 189–202).
